# Editorial: Circadian desynchrony: Consequences, mechanisms, and Open Issues

**DOI:** 10.3389/fphys.2023.1177643

**Published:** 2023-03-17

**Authors:** Roee Gutman, Julie S. Pendergast, Wataru Nakamura, Shihoko Kojima

**Affiliations:** ^1^ Laboratory of Integrative Physiology, The Department of Nutrition and Natural Products, MIGAL—Galilee Research Institute, Kiryat Shmona, Israel; ^2^ Department of Animal Sciences, Faculty of Sciences and Technology, Tel-Hai College, Upper Galilee, Israel; ^3^ Department of Biology, University of Kentucky, Lexington, KY, United States; ^4^ Department of Oral Chrono-Physiology, Graduate School of Biomedical Sciences, Nagasaki University, Nagasaki, Japan; ^5^ Department of Biological Sciences, Fralin Life Sciences Institute, Virginia Tech, Blacksburg, VA, United States

**Keywords:** circadian rhythm, misalignment, entrainment (light), molecular clock, Adaptive fitness, artificial light at night (ALAN)

## Introduction

### Studying circadian desynchrony

Most biological processes show sustained circadian rhythms generated by endogenous clocks oscillating with −24-h periods. In mammals, these endogenous rhythms are generated by a molecular timekeeping system, a transcriptional-translational feedback loop (Panda, 2016). In single-cell cyanobacteria, phosphorylation cycles generate endogenous 24-h rhythms (Nakajima et al., 2005). The circadian clock(s) detects and entrains to environmental cues called zeitgebers. Proper entrainment of endogenous rhythms to environmental cycles allows organisms to anticipate predictable changes in their environment and is associated with longevity, reproductive success, and fitness (Penev et al., 1998; Arble et al., 2010; Wyse et al., 2010; Gutman et al., 2011; Libert et al., 2012; Wyse, 2012; Steckler et al., 2016; Zhou et al., 2016).

There are many ways that circadian timing systems can be disrupted. Circadian desynchrony can be imposed in the laboratory by altering molecular timekeeping mechanisms or zeitgebers. Irregular changes in the environmental zeitgebers caused by human activities (e.g., light pollution, global warming, shift work, and mistimed eating) also impact circadian synchrony. The articles in this Research Topic address the consequences of these various models of circadian desynchrony.

### Impact of changes in environmental zeitgebers on physiology and behavior

The light-dark cycle is the most salient zeitgeber for many organisms. While circadian timing systems evolved in an environment with darkness at night, modern technology has altered the light-dark cycle such that exposure to artificial light at night is commonplace. Indoor lighting and using electronic devices at night affect the entrainment of the circadian system in humans (Wright et al., 2013; Chang et al., 2015). Moreover, chronic exposure to artificial light at night impacts metabolism and reproduction (Dominoni et al., 2016).

In this Research Topic, Rumanova et al. showed that nocturnal rats exposed for only 2 weeks to dim artificial light at night had increased cholesterol and disrupted daily rhythms of plasma metabolites. Bilu et al. reviewed studies similarly showing that artificial light at night and changes in photoperiod in a diurnal rodent, the fat sand rat (*Psammomys obesus*), exacerbated type 2 diabetes, obesity, adipocyte dysfunction, cataracts, depression, and anxiety. The authors also advocate using the diurnal fat sand rat to study the effects of modern lifestyles on health since, unlike most animal models, they have behavioral phenotypes that are more similar to humans than nocturnal rodents. Notably, increasing the salience of zeitgebers through interventions such as morning bright light exposure or access to running wheels also improved or prevented the development of health Research Topic. As such, Bilu et al. suggest implementing these interventions in clinical trials to determine if similar interventions could also positively impact human health.

Environmental zeitgebers are also altered in hospital settings. Hollis et al. examined whether patients in intensive care had impaired molecular circadian rhythms. Using RNA-seq datasets from the Genotype-Tissue Expression (GTEx) project, the authors found that hundreds of genes from multiple tissues were differentially expressed between patients in intensive care and those who died acutely. Interestingly, clock output genes, but not core clock genes, were overrepresented among these dysregulated genes in patients in intensive care, suggesting that the clock output mechanisms were affected. Notably, the gene expression profile of patients in intensive care was similar to previous clinical studies of sleep deprivation and fasting. Hollis et al. suggested that intensive care protocols that restore sleep/wake and nutritional rhythms may be of benefit.

The contribution of maintaining regular cycles of sleeping and eating has been demonstrated in dozens of studies, but adhering to regular schedules is challenging due to social and work obligations. The discrepancy between endogenous circadian timing and social and work obligations is called social jetlag (Wittmann et al., 2006). Social jetlag is associated with metabolic dysfunction and psychiatric disorders (Caliandro et al., 2021). Therefore, there is a need to identify ways to counteract social jet lag apart from maintaining regular eating and sleeping schedules. In this Research Topic, Oneda et al. found that wheel running exercise in mice improved resynchronization of body clocks in conditions that mimicked social jet lag. As with exposure to dim light at night, exercise could be an approach to improve social jet lag in humans Oneda et al.

### Impact of altering the function of molecular circadian clocks on behavior and adaptive fitness

The role of the molecular circadian clock in regulating fitness and behavior can be investigated in organisms with mutated circadian clock components. In this Research Topic, Zhao et al. studied a strain of cyanobacteria, Synechocystis, that has been a popular model for studies of photosynthesis and as a microbial fuel source, but whose circadian clock has not been well-studied. The authors developed a new luminescence reporter for Synechocystis, which allowed them to study the molecular timekeeping mechanisms of this cyanobacteria. They found that kai cluster mutants (kaiAB1C1-ko) that had severely disrupted rhythms were outcompeted by wild-type strains in mixed cultures.

A large body of evidence indicates that the hypothalamic suprachiasmatic nucleus (SCN) generates mammalian circadian rhythms in mice (Moore and Eichler, 1972; Stephan and Zucker, 1972). Neurons in different subdivisions of the SCN synthesize arginine vasopressin (AVP) and vasoactive intestinal polypeptide (VIP). A classical hypothesis is that the mammalian circadian system is composed of two mutually coupled oscillators, and these neuropeptides are molecular markers of these oscillators in the SCN. Peng et al. developed *Vip* ^tTA^ knock-in mice that express tetracycline transactivator (tTA) specifically in VIP neurons. This genetically engineered mouse provides an effective tool for validating a multi-oscillator circadian system that allows optimal entrainment, which is essential for environmental adaptation Peng et al.

In summary, the impacts of circadian desynchrony on health and fitness are vast. This is an important area of study as it may elucidate interventions for human health and for minimizing the impact of human activities on animals in the wild.
